# Books and Magazines

**Published:** 1906-06

**Authors:** 


### Books and Magazines.

A Treatise on Surgery—In two volumes. By George R. Fowler, M. D., Examiner in Surgery, Board of Medical Examiners of the Regents of the University of the State of New York; Emeritus Professor of Surgery in the New York Polyclinic, etc. Two imperial octavos of 725 pages each, with 888 text illustrations and 4 colored plates, all original. Philadelphia and London: W. B. Saunders Co., 1906. Per set: Cloth, $15, net; half Morocco, $17, net.

We have been looking forward to the appearance of this work with the greatest expectations, for Dr. Fowler's endeavors in the field of practical surgery have been such as to stamp his writings with unquestionable authority. It is not too much, indeed, we feel it is too little, to say that our expectations have been fully realized. The work is a masterpiece. It is an accurate, up-to-date treatise on surgery, skillfully presented. This entirely new work presents the science and art of surgery as it is practiced today. The first part of the work deals with general surgery, and embraces what is usually included under the head of principles of surgery. Special attention is given to the subject of inflammation from the surgeon's point of view, due consideration being accorded the influences of traumatism and bacterial infection as the predisposing and exciting causes of this condition. Then follow sections on the injuries and diseases of separate tissues, gunshot injuries, acute wound diseases, chronic surgical infections (including syphilis), tumors, surgical operations in general, foreign bodies, and bandaging. The second part of the work is really the clinical portion, devoted to regional surgery. Herein the author especially endeavors to emphasize those injuries and surgical diseases that are of the greatest importance, not only because of their frequency, but also because of the difficulty of diagnosis and the special care demanded in their treatment. Throughout special attention has been given to diagnosis, the section on laboratory aids being unusually excellent. The text is elaborately illustrated with entirely new and original illustrations, and evidently neither labor nor expense has been spared to bring this feature of the work up to the highest standard of artistic and practical excellence.

---

THE WORLD'S ANATOMISTS. By G. W. H. Kempner, M. D.; with 11 illustrations, 9 portraits. P. Blakiston's Son & Co., Philadelphia, 1906. Concise Biographies of Anatomic Masters from 300 B. C. to the present time, whose names have adorned the literature of the medical profession. Dr. Kempner is Professor of the History of Medicine in the Medical College of Indiana, at Indianapolis. Revised and enlarged from the original serial publications in the Medical Book News. Miniature reproductions of Rembrandt's famous "Demonstration in Anatomy," and portraits of Harvey, Hunter, Winslow, Heath, Holden, Morris, Vesalius and others embellish the text. Price, 50 cents.

---

PRE-NATAL CULTURE; SYSTEMATIC METHOD OF MOULDING THE TENDENCIES OF OFFSPRING BEFORE BIRTH. By A. E. Newton, author of "The Better Way." Stockholm Publishing Co., Inc., Chicago. Price not stated. 109 pages; paper cover.

A live book which parents should read.

---

A TEXT-BOOK OF MATERIA MEDICA, THERAPEUTICS, AND PHARMACOLOGY. By George F. Butler, Ph. G., M. D., Associate Professor of Therapeutics in the College of Physicians and Surgeons, Chicago. Fifth edition, thoroughly revised by Smith Ely Jelliffe, M. D., Ph. D., Professor of Pharmacognosy and Instructor in Materia Medica and Therapeutics in Columbia University (College of Physicians and Surgeons), New York. Octavo of 694 pages, illustrated. Philadelphia and London: W. B. Saunders Co., 1906. Cloth, $4, net; half Morocco, $5, net.

For this fifth edition Dr. Butler's text-book has been entirely remodeled, rewritten, and reset, bringing it in accord with the new (1905) Pharmacopeia. All obsolete matter has been eliminated, and special attention has been given to the toxicologic and therapeutic effects of the newer compounds. We notice with much satisfaction that the general arrangement of the book has been so changed that those drugs the predominant action of which is on one system of organs of the body are grouped together, thus suggesting their therapeutic as well as their pharmacologic alliance. We believe this classification to be more thoroughly practical and useful than any other. By use of a more compact type the work has been reduced in size. It is a pleasure to us to recommend this book to the profession, for it is no doubt the most thorough, and in every way the best on the subjects it includes.

---

A Reference Handbook of the Diseases of Children — For students and Practitioners. By Prof. Ferdinand Fruhwald, of Vienna. Edited, with additions, by Thompson S. Westcott, M. D., Associate Professor of Diseases of Children in the University of Pennsylvania. Octavo volume of 553 pages, with 176 illustrations. Philadelphia and London: W. B. Saunders Company, 1906. Cloth, $4.50, net; half Morocco, $5.50.

To those of the medical profession who are acquainted with Professor Fruhwald's work in the original German, it is hardly necessary to speak of the extremely valuable service the W. B. Saunders Company has performed in presenting this English translation. It must be said, however, that the translation possesses an advantage over the original—though it be the work of a leader amongst leaders in pediatric knowledge—in that the editor, Dr. Westcott, has incorporated much valuable matter, the results of his own valuable experience. With a view to making it of special service as a practical reference book, the individual diseases have been arranged alphabetically, with numerous cross-references. This is a novel feature of untold value to the busy practitioner who wishes information quickly. Special consideration has been given to symptomatology, and the prophylactic, therapeutic, and dietetic treatments have been elaborately discussed; especially is therapy treated according to the latest discoveries. The illustrations are practical and therefore excellent, nearly all being reproductions of original photographs and drawings representing cases from Professor Fruhwald's own clinic. Indeed, we can foresee for this work the same great success in this country that it has achieved in Germany.

---

NURSING ETHICS: FOR HOSPITAL AND PRIVATE USE. By Isabel Hampton Robb, Graduate of the New York Training School for Nurses attached to Bellevue Hospital; late Superintendent of Nurses and Principal of the Training School for Nurses, Johns Hopkins Hospital, Baltimore, Md.; late Superintendent of Nurses, Illinois Training School for Nurses, Chicago, Ill., etc. In one volume of 273 pp, size 5x7½ inches. Price, $1.50, bound in cloth. Sent postpaid on receipt of price by E. C. Koeckert, Publisher, 715 Rose Building, Cleveland, O.

This work, which represents an entirely new departure, is intended as a complement to the author's text-book on The Principles and Practice of Nursing. It emphasizes the importance of systematic instruction in the ethics of nursing in training-schools, while at the same time it will prove of material assistance to the unexperienced graduate nurse. No attempt has been made in it to lay down or even to suggest anything in the way of a formal code of ethics. The author has contented herself with a brief consideration of the nature of the ethical laws involved in the carrying on of nursing work and has tried to show in what way they may be made to have a practical bearing upon a nurse's duties and actions. At the same time the results of her long experience furnish texts upon which each superintendent of nurses may enlarge according to her knowledge and experience. The matter is arranged in a systematic and comprehensive manner. The main subjects considered are divided into twelve chapters, and deals in part with: Nursing as a Profession; Qualifications; The Probationer; The Junior Nurse, Methods of Instruction, Personality, The Face, Eye, Voice, Touch, Hearing, Walk, Sympathy, Manner, The Spirit of Nursing, Discipline; Health, The general care of the body, care of the hands and feet, etc.

THE OPERATING ROOM AND THE PATIENT. By Russell S. Fowler, M. D., Surgeon to the German Hospital, Brooklyn, N. Y. Octavo of 172 pages, fully illustrated. Philadelphia and London: W. B. Saunders Co., 1906. Cloth, $2, net.

In Dr. Russell Fowler's admirable work we have a book that has long been needed, one that to our knowledge is unique in that it is the only work on the market devoted entirely to operative technic, with the pre-operative procedures of sterilization and preparation. Written by a surgeon of rich clinical experience for the use of surgeons, nurses assisting at an operation, and hospital internes, it clearly describes the preparation of material of all kinds, indicates the instruments required for the various operations, details the preparation and care of the patient before and after operation, and the methods of anesthetization, describes and illustrates the position of the patient for different operations, and contains all other information a knowledge of which is necessary to produce the highest efficiency. Indeed, it is a most excellent and most valuable work for practical use, and the operating surgeon will find it of additional value as it furnishes him a guide to which he may readily add his own variations of technic.

Nursing in the Infectious Fevers. By George P. Paul, M. D., Assistant Visiting Physician and Adjunct Radiographer to the Samaritan Hospital, Troy, New York. 12mo of 200 pages, illustrated. Philadelphia and London: W. B. Saunders Co., 1906. Cloth, $1, net.

It is evident to us that Dr. Paul has written his book on Fever Nursing especially for the nurse and with a knowledge of the subject that can have been gained only by intimate association with routine hospital work. The care and management of each fever has been accorded special attention, as these subjects are of particular interest to the nurse. The author has divided his work into three parts: The first treats of fevers in general; the second of each fever individually; the third deals with practical procedures and information necessary to the proper management of the various diseases discussed, such as antitoxins, bacteria, urine examination, poisons and their antidotes, enemata, topical applications, antiseptics, weights and measures, etc. Altogether, it will be found that Dr. Paul has rendered a valuable service, not only to the nursing, but also to the medical profession, as much of the information given is not without the frequent needs of the general practitioner.

---

A Text-Book on the Practice of Gynecology—For Practitioners and Students. By W. Easterly Ashton, M. D., LL. D., Fellow of the American Gynecologic Society; Professor of Gynecology in the Medico-Chirurgical College of Philadelphia. Second edition, revised. Octavo of 1079 pages, with 1046 original line drawings. Philadelphia and London: W. B. Saunders Co., 1906. Cloth, $6.50, net; half Morocco, $7.50, net.

The fact that two editions of Dr. Ashton's new work have been required in the short period of six months indicates beyond a doubt that the medical profession was quick to appreciate the practical merits of the book; indicates that the general practitioner wants a treatise on gynecology that does not assume him to be an expert gynecologist, but rather describes in detail, not only what should be done in every case and emergency, but also precisely *how to do it.* Owing to the short time that has elapsed since the appearance of the first edition, and also from the thorough manner in which Dr. Ashton handled his subject originally, the changes in this edition are necessarily few in number and limited chiefly to the correction of a few typographic errors and the alteration of several of the illustrations. In reviewing this new edition we can not refrain from again speaking of the very practical illustrations. There are 1046 of them, all original line drawings made especially under Dr. Ashton's personal supervision, from actual apparatus, living models, dissections on the cadaver, and from the operative technics of other authors. All superfluous anatomic surroundings are eliminated and the operations and procedures are detailed step by step with a clearness and accuracy we have never before seen. Certainly, the success the work has won is well deserved and fully to have been expected.

